# Rotational knee laxity: Reliability of a simple measurement device in vivo

**DOI:** 10.1186/1471-2474-9-35

**Published:** 2008-03-18

**Authors:** Andrew G Tsai, Volker Musahl, Hanno Steckel, Kevin M Bell, Thore Zantop, James J Irrgang, Freddie H Fu

**Affiliations:** 1 Department of Orthopaedic Surgery, University of Pittsburgh, Pittsburgh, PA, USA

## Abstract

**Background:**

Double bundle ACL reconstruction has been demonstrated to decrease rotational knee laxity. However, there is no simple, commercially-available device to measure knee rotation. The investigators developed a simple, non-invasive device to measure knee rotation. In conjunction with a rigid boot to rotate the tibia and a force/moment sensor to allow precise determination of torque about the knee, a magnetic tracking system measures the axial rotation of the tibia with respect to the femur. This device has been shown to have acceptable levels of test re-test reliability to measure knee rotation in cadaveric knees.

**Methods:**

The objective of this study was to determine reliability of the device in measuring knee rotation of human subjects. Specifically, the intra-tester reliability within a single testing session, test-retest reliability between two testing sessions, and inter-tester reliability were assessed for 11 male subjects with normal knees.

**Results:**

The 95% confidence interval for rotation was less than 5° for intra-tester, test-retest, and inter-tester reliability, and the standard error of measurement for the differences between left and right knees was found to be less than 3°.

**Conclusion:**

It was found that the knee rotation measurements obtained with this device have acceptable limits of reliability for clinical use and interpretation.

## Background

The anterior cruciate ligament (ACL) is the primary restraint to anterior tibial translation, secondary restraint to valgus rotation, and tertiary restraint to internal tibial rotation [1]. Rotational knee laxity may predict later osteoarthritis [2,3] and is closely correlated with patient discomfort [4]. Though reduced after single-bundle ACL reconstruction [5], rotational laxity may still exist, even though the anterior translation has been adequately restored [6,7]. Anatomic double-bundle reconstruction, in contrast, replaces the anteromedial (AM) bundle as well as posterolateral bundle (PL) [8,9]. Anatomic ACL reconstruction may better restore normal knee kinematics in six degrees of freedom (6-DOF) [10-13]. Diagnosis of rotational knee laxity after ACL tears in the office is based on patient history and subjective un-instrumented physical examination. Concomitant injury to other ligamentous structures can lead to false positives, and patient guarding can reduce the sensitivity of tests and lead to false negatives.

### Un-Instrumented Physical Exams

Un-instrumented physical examination is the gold standard for assessing knee ligamentous injuries, although these exams are subjective and dependent on examiners skill and experience. The Lachman test, which is performed with the knee at 30° of flexion, is the most sensitive test [14]. At this flexion angle, the PL bundle is starting to become tight and is the primary restraint to anterior tibial translation. A difference of >3 mm in anterior tibial translation as compared to the uninjured, contralateral knee as well as a soft endpoint indicate a positive Lachman test, which is indicative of injury to the ACL, both in the AM and PL bundles [15]. The less-sensitive anterior drawer test is tested with the knee at 90° of flexion. When performing the anterior drawer test, the examiner draws the proximal tibia forward and uses his thumbs to palpate the tibiofemoral step off; the test is repeated with the foot in neutral, internal, and external rotation. The quality of the end point as well as the difference in translation between the patient's injured and uninjured knees indicates damage to the ACL, particularly the AM bundle, as well as secondary supports [16]. The Slocum test is similar to the anterior drawer test except it tests for rotational laxity and is performed with the foot and tibia internally rotated 30° and with the tibia externally rotated 15° [16,17]. The internal and external rotation tightens up the lateral and medial ligamentous structures respectively. A positive Slocum test is indicative of anterolateral or anteromedial laxity. The pivot-shift test is the most specific test for ACL injury and is oftentimes only testable during examination under anesthesia (EUA). To administer the pivot-shift test, the tester rotates the patient's tibia inward while the knee is flexed at 30° [18-20]. The tester then extends and subsequently flexes the knee. If a pivot shift is present, the examiner should feel an anterior subluxation of the knee during extension and a glide, clunk, or gross reduction during flexion, corresponding to grades I, II, or III, respectively [21,22].

### Instrumented Physical Exams

There are several commercially-available arthrometers used clinically to quantify anterior-posterior knee laxity, including the KT-1000 (MEDmetric Corporation, San Diego, CA), Rolimeter (Aircast, Summit, NJ), Acufex Knee Signature System (Acufex Inc., Mansfield, MA), and Stryker Knee Laxity Tester (Stryker Corporation, Kalamazoo, MI) [16,23,24], though the KT-1000 is the most widely used [16]. The Rottometer [25] and Vermont Knee Laxity Device [26] may also be used to determine rotational knee laxity. The Rottometer is a modified chair design with the foot strapped down to a plate; knee rotation is measured with a goniometer. The Rottometer is a simple, easy-to-use device; however, it is not portable and is not capable of measuring off-axis loading. The Vermont Knee Laxity Device is a large, complex device that is capable of accurately measuring knee kinematics and simulating various loading situations. Its size, however, prohibits it from being portable and being used in the office setting. A simple device that measures rotation in a non-invasive manner is the Lars Rotational Laxiometer [27], which is strapped externally to the subjects' tibia. The Lars Rotational Laxiometer, however, is not able to measure the moment applied by the observer during testing and is unable to cancel out coupled motion of the femur; both were suggested as deficiencies by Bleday, et al. Additionally, computer assisted surgery (CAS) devices make it possible for a surgeon to accurately measure kinematics of the knee, but they are costly, complex, and require the patient to undergo surgery and thus can not be used for clinical follow-up [28,29].

There is no simple, commercially available device to measure knee rotation [30,31]. In a previous study, a simple device for non-invasive measurement of rotational laxity was described [32]. This device has been shown to have acceptable levels of test re-test reliability to measure knee rotation in a best case scenario in cadaveric knees. Therefore, the objective of this study was to determine the reliability of the new device to measure knee rotation in human subjects with normal knees. Specifically, the intra-tester reliability within a single testing session, the test-retest reliability between two testing sessions, and the inter-tester reliability were evaluated. It was hypothesized that knee rotation measurements obtained with the new device will have acceptable limits of reliability for clinical use and interpretation.

## Methods

The rotational knee laxity measurement device consists of an Aircast^® ^Foam Walker (Aircast, Summit, NJ) fitted with a 6-DOF universal force/moment sensor (UFS-Model 4015; JR3 Inc. Woodland, CA) and is used in conjunction with a Nest of Birds (NOB, Ascension Technologies, Inc. Burlington, VT) magnetic tracking system [32]. The 6-DOF universal force/moment sensor (UFS) is affixed to the sole of the Aircast boot with metal plates and screws. Another metal plate and a handle bar are attached to the front of the load cell, and a liquid-filled bubble-level is secured to the bar for the purpose of aligning the load cell to the ground. The magnetic tracking system consists of a compact NOB chassis, 4 magnetic sensors, and a transducer. The sensors and the transducer communicate with the chassis via cables. A computer system is used to collect motion data from the NOB sensors and monitor the forces and moments of the UFS. Matlab (The Mathworks, Natick, MA), a mathematical computing software package, is used to interface with the magnetic sensors and the UFS from the computer.

The study was conducted on human subjects with the prior, written consent of the subjects and the approval of the University of Pittsburgh Institutional Review Board (#0505098). There were 11 males between the ages of 27 and 35, with a mean age of 30.3 years. The subjects had an average height of 187 cm and an average mass of 89.5 kg. The shoe size of the subjects was uniform, between 10.5 and 13 (US men's) with an average of 11.5. Therefore, a medium sized Aircast Foam Walker was chosen to provide snug fit for all subjects. See Table 1 for more details on subjects. Prior to testing with the knee laxity measurement device, each subject underwent examination according to IKDC protocol [21] to ensure that subject had normal knees bilaterally. The examinations included manual Lachman and anterior drawer tests, instrumented Lachman test with Rolimeter, pivot shift test and manual dial test. All subjects were determined knee normal by manual examination (Table 2).

**Table 1:** Subject Statistics

**Subject**	**Age**	**Height (cm)**	**Shoe size (US)**	**Weight (kg)**	**BMI**
**1**	31	188	12	88.5	25
**2**	30	188	12	86.2	24.4
**3**	34	196	13	88.5	23.1
**4**	35	191	12	83.0	23.1
**5**	28	188.0	10.5	90.7	25.7
**6**	29	188.0	13	104.3	29.5
**7**	27	185	10.5	86.2	25.1
**8**	31	180	11	88.5	27.2
**9**	27	178	10.5	86.2	27.3
**10**	31	196	13	93.0	24.3
**11**	29	180	11	88.5	27.2

Table 1 shows age, height, shoe size, weight, and BMI of all subjects.

**Table 2:** Manual Examination Data of Subjects Prior to Test (all subjects tested same bilaterally)

				**Manual Dial Test**			
				
**Subject**	**Manual Lachman (mm)**	**Instrumented Lachman with Rolimeter (mm)**	**Pivot Shift**	**internal 30°**	**external 30°**	**internal 90°**	**external 90°**
**1**	1	2	normal	10	20	10	20
**2**	1	2	normal	10	15	10	15
**3**	1	3	normal	10	10	10	15
**4**	1	2	normal	10	20	10	20
**5**	1	2	normal	10	15	15	20
**6**	1	2	normal	5	10	5	10
**7**	1	1	normal	5	5	5	5
**8**	1	1	normal	5	10	5	10
**9**	1	1	normal	10	15	15	20
**10**	1	2	normal	10	15	15	20
**11**	1	2	normal	10	15	10	15

Table 2 shows results from manual examination prior to testing the subjects with the new device. Data include manual Lachman, instrumented Lachman with a Rolimeter measurement device, the pivot shift test, and manual dial test. Manual dial test was tested at 30° and 90° and the data represent results for internal and external rotation. All subjects tested the same bilaterally.

**Figure 1:**
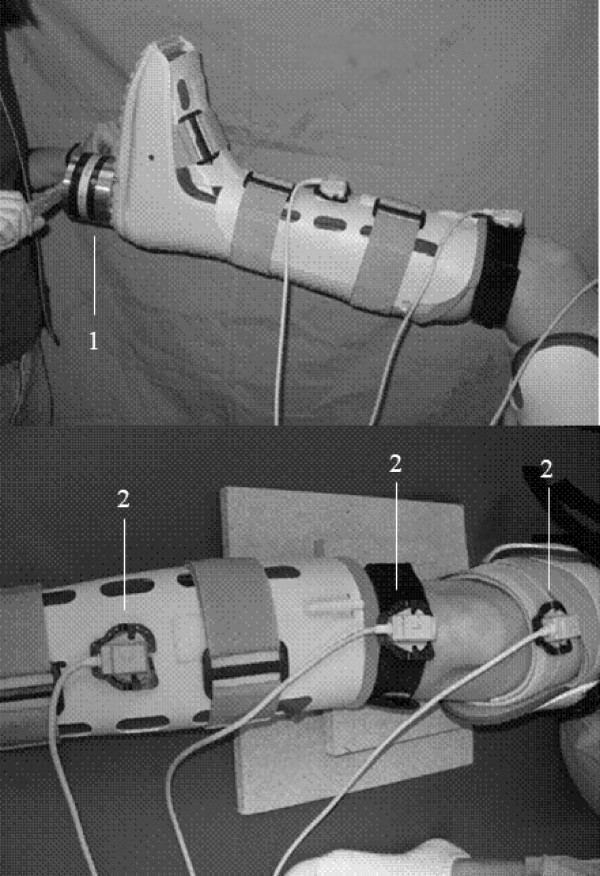
**Knee Laxity Measurement Device**. This picture depicts the side view and anterior view of the Knee Laxity Measurement Device in use. Shown are the 6-DOF universal force/moment sensor and handle bar (1) attached to the Aircast boot. The three magnetic trackers (2) are attached to the boot, tibia, and femur to record measurement of rotational knee laxity.

**Figure 2:**
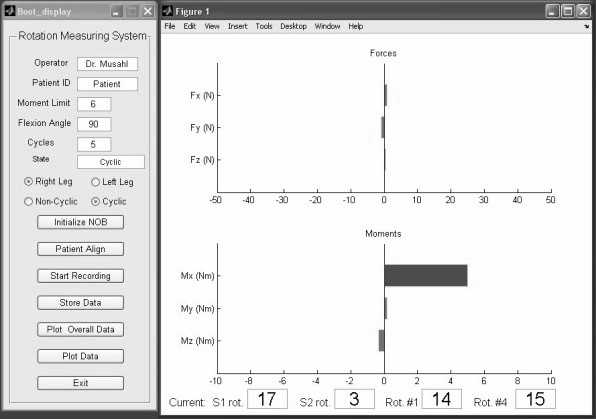
**Screen-shot from Software Interface**. The computer software interface displays the forces and moments for 6 degrees of freedom. Tibial rotation is also displayed. The visual feedback, as well as audio feedback, assists the investigator in achieving a consistent moment while reducing off-axis loading.

For application of the device on a human subject, the air cells inside the Aircast Foam Walker were inflated to a gauge pressure of 40 mmHg prior to each test. The subject, who was lying supine on an examination table, confirmed a snug fit of the device without being uncomfortable. The NOB is capable of tracking the position and orientation of the tibia, femur, and boot simultaneously. Placement of the magnetic sensors on the subject's leg is accomplished with hook and loop fasteners attached to fabric straps. The magnetic sensors were placed on specially marked areas on the anterior surface of the Aircast boot, the medial surface of the proximal tibia 1 cm distal from the tibiofemoral joint line, and the anterior surface of the thigh. The actual placement of the sensors was indicated on the skin with a non-permanent skin marker for the purpose of determining if the magnetic tracker had been severely disturbed between tests. A handle bar mounted on the UFS allowed the tester to rotate the boot easily as well as support the lower leg at the appropriate angle of knee flexion, and a bubble-level mounted on the handle bar allowed the tester to achieve a consistent starting point and sensor alignment with respect to the ground (Figure 1). A section of 2" polyvinyl chloride pipe (PVC) mounted vertically on the side of the examination table prevented each subject's hip and leg from externally rotating through the hip joint during examination. Additionally, during the 30° knee flexion tests, a raised, support platform was placed under the thigh to angle the leg and allow the tester to more easily maintain the proper flexion angle of the knee. The software interface (Figure 2) and computer provided useful audio-visual feedback to the tester to indicate when moments of 2, 4, and 6 Nm had been measured at the load cell. At 2, 4, and 6 Nm of torque, tones with increasingly higher frequencies played through the speakers as a guide for the tester. Also at 6 Nm, the graph displayed on the screen changed from green to red as a visual indication that the target load was achieved.

The relative motion of the tibia with respect to the femur is considered internal and external rotation of the knee and is the method by which the measurement device operates. The system utilizes a Cardan angle sequence based on Grood and Suntay conventions [33], and the coordinate system is aligned with the long axis of the tibia through the bubble level. Two of the three rotation angles are always held constant during testing, so this methodology allows repeatable, efficient readings consistent with current manual testing without adding to the testing time or the requisite technical knowledge of the clinician. Because the device uses three sensors, it can more faithfully record motions of the tibia. When the observer is ready to begin the test, he aligns the boot position level to the ground as indicated by the bubble level. The computer accepts the aligned position as the starting position, and then the observer performs cycles of internal and external rotation. The initial position of the tibia sensor relative to the femur sensor is then compared with the relative position for each subsequent sample. By this method, undesired femoral motion (for example, hip rotation) is able to be mathematically eliminated from coupled tibia motion to give only the relative motion of the tibia to the femur. Additionally, the sensor attached to the boot allows the load cell to account for changing orientations of the boot, which may be slightly different from those of the subjects' tibia. By being able to see the loads on the computer screen, the tester is able to see if unintended off-axis loading is being applied.

Three aspects of the device were tested in determining the reliability of the device. The first was intra-tester reliability within a single testing session. During each trial, at 90° and 30° of knee flexion, the examiner took five measurements for internal-external rotation, and the reliability within those five trials was observed. Another aspect was test-retest reliability. To gauge test-retest reliability, a single examiner removed the device after measuring the internal-external rotation, and, a short time later, replaced the device and re-measured the internal-external rotation. The final aspect was the inter-tester reliability. Inter-tester reliability was measured by comparing the measurements of a secondary examiner with those of the primary examiner.

Statistical analysis was performed using Statistical Package for the Social Sciences (SPSS Inc., Chicago, IL). To assess intra-tester, test re-test and inter-tester reliability, intraclass correlation coefficients (ICC) were calculated. The ICC was calculated with formula 2,1 for single measures agreement (for intra-tester reliability) and ICC formula 2,5 for average measures agreement [34]. The ICC value was further used to estimate the standard error of measurement (SEM), which is defined as the standard deviation of the measurements multiplied by the square root of one minus the ICC. The 95% confidence intervals for the standard error of measurement are calculated as (1.96 * SEM) and are interpreted as the degree of uncertainty of an individual's observed measurement [35].

## Results

There were no adverse effects noted by the subjects, specifically there were no neurovascular symptoms, no skin irritation, and no adverse effects regarding the amount of applied moment. The total testing time per subject was approximately 20–25 minutes. The pressure in the air cells of the boot was monitored constantly and was consistent between tests, as long as the boot was not removed. Therefore, the air cells were deflated and re-inflated to 40 mmHg whenever the boot was removed or adjusted.

Data for all subjects is summarized in Table 3. The total axial rotation for N = 22 knees was 18.5° ± 4.7° at 90° of knee flexion and 25.8° ± 5.9° at 30,° of knee flexion. For intra-tester reliability within one testing session with knee flexion of 30° and 90°, the single measure ICC's were both >0.95, with associated SEM's <1° and a 95% confidence interval's <2.0°. For inter-tester reliability at 90° of knee flexion (n = 21, data from 1 knee was lost), the average measures ICC and SEM were 0.88 and 1.6°, respectively, resulting in a 95% confidence interval of 3.2°. At 30° of knee flexion (n = 22), the average measures ICC and SEM for inter-tester reliability were 0.81 and 2.6° respectively, yielding a 95% confidence interval of 5.1°. For 90° of knee flexion, the average measures ICC and SEM for test-retest reliability were 0.77 and 1.9°, respectively, for a 95% confidence interval of 3.8°.

**Table 3:** Results on rotational laxity

	90°, observer 1, time 1	90°, observer 2, time 1	30°, observer 1, time 1	30°, observer 2, time 1	90°, observer 1, time 2
Subject 1, right	17.9	16.0	27.6	33.5	23.4
Subject 1, left	15.5	14.3	27.1	34.8	16.9
Subject 2, right	19.1	16.0	22.2	32.2	16.3
Subject 2, left	24.4	20.4	27.8	30.7	17.0
Subject 3, right	17.2	--	20.4	25.9	16.6
Subject 3, left	16.7	18.9	25.1	32.5	15.8
Subject 4, right	14.6	17.0	17.6	29.1	15.7
Subject 4, left	16.3	20.2	22.6	30.3	15.76
Subject 5, right	13.5	19.1	38.3	39.1	13.7
Subject 5, left	11.3	13.8	37.5	40.3	14.0
Subject 6, right	15.5	17.8	19.4	22.3	20.2
Subject 6, left	12.1	16.7	22.4	21.7	16.2
Subject 7, right	26.8	26.4	25.9	27.9	21.6
Subject 7, left	18.1	17.9	15.5	18.4	19.8
Subject 8, right	17.0	21.0	37.0	38.4	20.0
Subject 8, left	26.6	32.4	28.5	29.3	24.2
Subject 9, right	26.6	26.0	28.1	30.2	21.9
Subject 9, left	25.4	28.8	26.0	28.5	22.7
Subject 10, right	20.1	20.0	27.1	31.5	16.0
Subject 10, left	15.1	20.3	23.8	27.2	14.6
Subject 11, right	20.0	21.4	23.5	26.7	21.2
Subject 11, left	18.3	19.3	23.9	24.0	24.4

Table 3 shows the data for all subjects, both left and right knees. Column 1 of the data shows rotation at 90° of knee flexion for observer 1 at time 1. Column 2 shows rotation at 90° for observer 2 at time 1. Columns 3 and 4 are the data for observers 1 and 2 at time 1 at 30° of knee flexion. Column 5 is the data for 90° of knee flexion for observer 1 at time 2.

The average side to side difference in total internal-external rotation of the tibia between normal knees was 3.5°. To estimate the magnitude of the differences to be expected between normal knees, we used the ICC to calculate the level of agreement in total tibial rotation between the right and left sides as well as the SEM. The ICC's were all greater than 0.75 and the SEM's were all less than 2°.

## Discussion

In the late 1960's and early 1970's, rotatory laxity of the knee as a consequence of ACL tears began to receive attention [17]. Clinical tests for assessment of rotational laxity, such as the pivot shift test or the Losee test were subsequently developed [16,17,30,36]. Assessment of rotational laxity has now become standard practice in most institutions, highlighting the need for standardization and consistent documentation. Daniel et al. developed an instrumented test for measurement of anterior-posterior knee laxity in the 1980's [15], which has since become the gold standard for reporting outcomes of ACL reconstructions [37-39]. Currently, there are efforts being made to develop instrumented tests for rotational knee laxity [25,28,40]. With the concept of anatomic ACL reconstruction, there is a demand for consistent documentation of rotational laxity. The objective of the present study was to determine the reliability of a simple device, previously validated in vitro [32], for measuring knee rotational laxity in vivo. Such a device should yield measurements of knee laxity that are consistent during a single testing session for a single tester, consistent for a single tester over multiple testing sessions, and consistent between testers. Therefore, intra-tester reliability, test-retest reliability, and inter-tester reliability were calculated in order to gauge reliability of the system. The results support the hypothesis that the device has acceptable limits of reliability, and specifically, repeatability of the device was found to be within 5° of rotation. The highest inter-tester reliability was observed at 30°, which the authors believe may be attributed to excessive hip and thigh rotation during testing. The clinical significance of 5° of measurement error when measuring differences between injured and non-injured knees or within an injured knee over time before and after treatment has yet to be determined. We currently have ongoing studies that are designed to determine if the ability to detect differences of 5° of tibial rotation is clinically meaningful in patients following ACL, PCL and posterolateral corner injury and surgery.

Determining the reliability of the device was performed with calculations of ICC and SEM. ICC is used as a measure of consistency between measurements, such as between two different observers. The SEM and associated 95% confidence interval are estimations of measurement precision of the device. For example, if the measured rotation of an individual is 15°, and a 95% confidence interval of 3.2° was calculated previously, as was the case for inter-tester reliability at 90° of flexion, then one can conclude that 95 times out of 100 the individual's true score lies within ± 3.2° of the measured value. Thus, if the individuals measured score was 20°, the 95% confidence interval for the individual's true score is 16.8° to 23.2°.

In the case of trauma to the knee, manual testing by a clinician can help diagnose specific ligamentous injury. Manual testing of rotational laxity of the knee, such as with the pivot-shift test, is highly variable and dependent on the tester's experience and ability to detect abnormal knee motions. The shift is described as a combined posterior translation and/or external rotation, but the presence of the two motions is inconsistent between subjects [5]. Instrumented clinical tests, such as the instrumented Lachman and anterior-drawer tests performed with the KT-1000 are sufficient in measuring anterior-posterior translation but cannot gauge rotational laxity. Tests performed with rigidly fixated electromagnetic sensors [5] or computer navigation [28] can accurately measure rotational kinematics, but such tests are invasive and impractical in the office setting. A simple-to-use, clinically-relevant device for rotational laxity measurement has yet to become commercially available.

Studies have shown that rotational knee laxity is highly variable between subjects and can range anywhere from 20° to 65° of rotation depending on flexion angle and testing method, so a reliable instrument for measuring knee laxity would be needed to detect abnormal rotational motion [25,41-43]. In a recent in vivo study [25], a similar external measurement device, the Rottometer, was compared to RSA results and found to have systematic error of around 100%. At 90° of flexion and 6 Nm of torque, the Rottometer device recorded 21° and 27° of internal and external rotation, respectively, while RSA recorded 10° and 16°, respectively. That error seems to arise because measurements were made at the foot without taking into account ankle rotation. Our results of 18.5° total rotation may understate the rotation because of skin and soft tissue artifacts. Though accuracy can still be improved, reliability of the device has been demonstrated within the testing parameters.

The new device presented in this study has potential use in clinic because it is portable, non-invasive, and requires no specific skills to operate. The ability to measure knee laxity with a simple device could be advantageous to both patients and physicians. Its ability to measure rotational laxity, versus other devices that measure anterior-posterior translation, can enhance a physician's ability to objectively assess knee injuries by complementing current tests. Rotational laxity may be present even in the absence of abnormal translational movements after single bundle ACL reconstruction. Therefore, during surgical reconstruction of the ACL, addition of the PL bundle may correct the rotational laxity [10-13], but measurement of the rotation still remains an issue.

The device and methodology presented were originally adapted from a prior, cadaveric study [32]. The investigators also conducted a pilot study involving volunteer subjects and used the results from the pilot study to improve the device and test procedure for the current study, since the results obtained during the pilot study were inconsistent. The pilot study yielded a test-retest ICC of 0.73 and a 95% confidence interval of 15° for total rotation. Based on the results, the investigators found it necessary to standardize the placement of the electromagnetic trackers to ensure better results from one test to another. Other adjustments were made to the device and protocol based on specific outcomes of the pilot study. The pilot study varied the pressure in the air cells from 0 to 40 mmHg in order to identify the importance of air cell pressure; pressure in the air cells was found to significantly influence the results of the trials. Pressure was, therefore, carefully monitored during this experiment to ensure the cells were inflated to a consistent pressure of 40 mmHg between trials. Also during the pilot study, the relative movement of the boot versus the tibia was monitored, and it was found that the boot moved around 30° relative to the tibia. To improve repeatability of the device, a medium sized boot was used in this study, versus a large sized boot in the pilot, which reduced movement between the subject's foot and the boot. The large boot fits male patients with shoe sizes from 10–13 according to the manufacturer, but it was found that the medium boot, which the company suggests for shoe sizes 7–10, gave a more snug fit and was not uncomfortable. Other changes made between the two studies included placement of the NOB transmitter. To improve accuracy of the magnetic tracking system, the transmitter was monitored to ensure it was placed between 10–24 inches from the sensors [44]. Finally, improvements were made to the computer's user interface to increase the visibility of the readout from the UFS (Figure 2). The purpose of the improved display was to allow the tester to compensate for incidental off-axis loading and allow the tester to more precisely control the amount of force on the subjects' knees. All the improvements appear to have improved the overall reliability of the device.

Limitations of this study include accuracy and consistency of measured bone movement due to skin artifacts. Though the Aircast boot locks the ankle in place and the air cells limit the movement of soft tissue, skin artifacts may diminish the device's ability to measure the actual movement of the tibia relative to the femur at the knee [45]. Because the magnetic trackers are attached with fabric strips to the surface of the skin on the femur and thigh, movement of skin, fat, or muscle around the bone is a source of error. Attaching the magnetic sensors to the most bony or muscular portions of the leg may provide the most reliable results. Since the measurements are taken from the femur and tibia, associated hip, boot, and ankle movements during the knee rotation, should not alter the knee laxity measurements. Also, since the device depends on the NOB magnetic tracking system for measurements, the limitations of the NOB become limitations of the device. The accuracy of any magnetic tracking system is sensitive to ferrous materials and interference from electromagnetic devices in the general vicinity, therefore, care should be taken to minimize interference from metallic or electromagnetic sources. The results of repeatability may have been affected since the investigators marked the subjects' skin with a marker to indicate the placement of the magnetic trackers.

## Conclusion

In conclusion, the new device appears to be repeatable within 5° of rotation in subjects with a normal knee. The new device is simple, portable, and easy to use. Future studies are planned to address the effectiveness of the device on patients with unilateral chronic ligamentous insufficiency to determine the device's ability to distinguish between normal and injured knees.

## Competing interests

The author(s) declare that they have no competing interests.

## Authors' contributions

AT assisted with experimental methods, subject testing, data analysis, and drafting of the manuscript. VM conceived of the study, acted as primary physician conducting testing, recruited subjects, analyzed results, and helped draft the manuscript. HS assisted in testing of subjects as a secondary observer and drafting the manuscript. KB assisted with study design, data acquisition, and drafting of the manuscript. TZ participated in initial design of the device and the study, pilot study testing, and drafting of the manuscript. JI assisted with designing the study, statistical analysis, and drafting of the manuscript. FF assisted with study conception and drafting of the manuscript. All authors read and approved the final manuscript.

## Pre-publication history

The pre-publication history for this paper can be accessed here:


